# Genomic data of an environmental *Escherichia coli* isolate shows high resemblance to *E. coli* K-12 reference strain MG1655.

**DOI:** 10.1016/j.dib.2021.107586

**Published:** 2021-11-18

**Authors:** Nicola Holden

**Affiliations:** a SRUC, Department of Rural Land Use, Craibstone Estate, Aberdeen AB21 9YA, UK; b Cell and Molecular Sciences, James Hutton Institute, Dundee DD2 5DA, UK

**Keywords:** Phylogenomics, Clonality, Whole genome alignment, Whole genome sequencing

## Abstract

*Escherichia coli* species exhibits a high genomic diversification from evolution, mobile genetic elements and recombination. An environmental *E. coli* isolate, ‘JHI_5025’ from a crop trial appeared to be clonally related to the historical reference isolate *E. coli* K-12 strain ‘MG1655’, warranting further genomic analysis. Their genomes share an average nucleotide identity of 99.74% and whole genome alignment showed little rearrangement of the JHI_5025 sequence compared to the reference. Five genomic islands not in the reference aligned to other sequences in the *Enterobacteriaceae*. Isolate JHI_5025 contained *E. coli* K-12 F plasmid sequence and at least one complete prophage sequence. The genome and comparison dataset provides utility of *E. coli* JHI_5025 as a representative contemporary genetic mimic of a well-known and much used workhorse strain.

## Specifications Table

**Table:** 

Subject	Omics: Genomics
Specific subject area	Phylogenomics comparative dataset of an environmental *E. coli* with reference strain *E. coli* K-12 MG1655
Type of data	Table Figure
How data were acquired	Bioinformatics tools: CLC Genomics Workbench (v20.0.4) with Whole Genome Comparison plugin installed (Qiagen Ltd.), run on a Windows 10 laptop computer (Core i5). Online tools for ANI, plasmid and phage sequences (detailed in Materials & Methods).
Data format	Raw Analyzed
Parameters for data collection	Isolate *E. coli* JHI_5025 from a collection of environmental *Escherichia* species isolates had an identical sequence type to the reference, *E. coli* K-12 strain MG1655. Experimental work showed functional differences (Marshall et al. 2016) and together with geographical and temporal separation warranted a detailed comparative analysis between the isolates.
Description of data collection	Genome sequence files were used for the comparison using bioinformatics tools. The genome sequence of *E. coli* JHI_5025 is complied from short-read technology (Illumina platform) in 71 contigs, in fasta format, which is categorised as ‘unfinished’. The reference genome is complete and well described (Blattner et al. 1997; Hayashi et al. 2006), with the most recent version (U00096.3) used in the comparison.
Data source location	Secondary data analysis using Primary data. Isolate collection from: James Hutton Institute, Dundee, UK Latitude and longitude for collected samples/data: 56•455 (lat) by −3•075 (lng) Whole genome sequence of *E. coli* isolate JHI_5025 accession number SAMEA104314548, sample alias E8. ENA Browser (ebi.ac.uk). Sequencing project number PRJEB22630.
Data accessibility	With the article AND: Repository name: European Nucleotide Archive JHI_5025 Data identification number: SAMEA104314548, sample alias E8 Direct URL to data: ENA Browser (ebi.ac.uk) MG1655 Data identification number: U00096.3 Direct URL to data: ENA Browser (ebi.ac.uk)

## Value of the Data

• An environmental *E. coli* isolate (soil, Scotland, 2009) was genetically highly similar to the *E. coli* K-12 reference strain MG1655, a historical isolate (neonate, USA, 1922) widely known as the ‘laboratory workhorse’
• The main beneficiaries are those using *E. coli* strain MG1655 genomic or functional data
• *E. coli* isolate JHI_5025 represents a contemporary genetic mimic of historical strain MG1655 enabling comparative analyses in basic and applied microbial sciences

## 1 Data Description

Environmental *Escherichia* species collected from a crop trial [4] were whole genome sequenced as part of a larger study [5]. Pan-genome analysis indicated clonality between isolate JHI_5025 and reference *E. coli* isolate MG1655 [2,3] despite functional differences [1], warranting detailed secondary analysis.

**Fig. 1:**
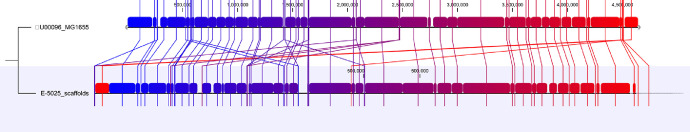
Whole genome alignment between JHI_5025 and the MG1655. Blocks of sequence are alignment with respect to each other, coloured by reference position. Numbering is supplied for genomic location relative to the reference sequence (MG1655: U00096.3).

The short-read (unfinished) genome sequence of JHI_5025 contains 4,887,055 nt in 71 contigs, G/C content of 50.5% and N50 of 265,259, and complete genome of strain MG1655 contains 4,641,652 nt with G/C content of 50.8% [3]. The average nucleotide identity between the strains is 99.74% (SD: 1.03%). Whole genome alignment showed little rearrangement (Fig. 1), with alignment of ‘gene’ features in 3,997,226 nt (JHI_5025) and 3,958,211 nt (MG1655). Inclusion of genome features aligned 4,578,512 nt (93.69% total genome) of JHI_5025 and 4,494,985 nt (96.84% total genome) of MG1655. Five unique ‘genomic islands’ occurred in JHI_5025 with respect to MG1655 (Table 1A), which matched sequences in *Yersinia pseudotuberculosis* (strain FDAARGOS_582), *E. coli* (strain RHBSTW-00046), *Enterobacter hormaechei* subsp. *steigerwaltii* (strain ME-1), *E. coli* O141:H4 (strain P13-6) and *E. coli* (NCTC9102), and one prophage (Entero_IME10). One plasmid was detected that mapped in three contig positions to *E. coli* K-12 plasmid F (accession AP001918) (Table 1B). 12 prophage regions were detected including an intact sequence absent in strain MG1655 (Table 1C) and two incomplete regions also on ‘genomic islands’ (Table 1A). Recognised recombination ‘hotspots’ *rbf* and *fim* gene clusters [6] were conserved, although flagella types differed (H39 for isolate JHI_5025, H48 for MG1655, Table 1D).

**Table 1:** Genomic features in *E. coli* isolate JHI_5025.

A. `Genomic Islands' (unique with respect to MG1655)									

Name	Contig	blastn_tophit	Accession number	Bit_Score (raw)	E_value	Query Length	Identities	Gaps	

island2	NODE_1_length_624063_cov_18.6826_ID_962 gap2	Escherichia coli strain RHBSTW-00046 chromosome	CP056894.1	16106 (17861)	0	27134	8938/8943 (99%)	0/8943 (0%)	
island2	NODE_1_length_624063_cov_18.6826_ID_962 gap2	Escherichia coli strain RHBSTW-00046 chromosome	CP056894.1	8592 (9528)	0	27134	4770/4774 (99%)	0/4774 (0%)	
island2	NODE_1_length_624063_cov_18.6826_ID_962 gap2	Escherichia coli strain RHBSTW-00046 chromosome	CP056894.1	17183 (19056)	0	27134	9528/9528 (100%)	0/9528 (0%)	
island2	NODE_1_length_624063_cov_18.6826_ID_962 gap2	Escherichia coli strain RHBSTW-00046 chromosome	CP056894.1	6855 (7602)	0	27134	3801/3801 (100%)	0/3801 (0%)	
island3	NODE_1_length_624063_cov_18.6826_ID_962 gap3	Enterobacter hormaechei subsp. steigerwaltii strain ME-1 chromosome	CP041733.1	2477 (2746)	0	39903	1555/1671 (93%)	9/1671 (0%)	
island3	NODE_1_length_624063_cov_18.6826_ID_962 gap3	Enterobacter hormaechei subsp. steigerwaltii strain ME-1 chromosome	CP041733.1	26619 (29520)	0	39903	16633/17877 (93%)	51/17877 (0%)	
island3	NODE_1_length_624063_cov_18.6826_ID_962 gap3	Enterobacter hormaechei subsp. steigerwaltii strain ME-1 chromosome	CP041733.1	3486 (3865)	0	39903	2297/2537 (91%)	16/2537 (0%)	
island3	NODE_1_length_624063_cov_18.6826_ID_962 gap3	Enterobacter hormaechei subsp. steigerwaltii strain ME-1 chromosome	CP041733.1	2166 (2401)	0	39903	1316/1393 (94%)	0/1393 (0%)	
island3	NODE_1_length_624063_cov_18.6826_ID_962 gap3	Enterobacter hormaechei subsp. steigerwaltii strain ME-1 chromosome	CP041733.1	1098 (1217)	0	39903	1113/1440 (77%)	12/1440 (0%)	
island1	NODE_19_length_84137_cov_23.9239_ID_1485 [R] gap1	Yersinia pseudotuberculosis strain FDAARGOS_582 chromosome	CP033711.1	25755 (28562)	0	15700	14287/14291(99%)	0/14291 (0%)	
island5	NODE_4_length_375183_cov_20.3356_ID_3592 [R] gap5	Escherichia coli strain NCTC9102 genome assembly	LR134227.1	11307 (12539)	0	6277	6274/6277 (99%)	0/6277 (0%)	
island4	NODE_6_length_265259_cov_20.0908_ID_899 gap4	Escherichia coli O141:H4 strain P13-6 chromosome	CP080223.1	17232 (19110)	0	9555	9555/9555 (100%)	0/9555 (0%)	
island6	NODE_9_length_131940_cov_22.2706_ID_1056	incomplete Blastn hits / PHAGE_Entero_IME10	NC_019501	n/a	n/a	38335	n/a	n/a	
B. Plasmid detection									

Position	Contig	Plasmid	Accession number	Identity	Position in contig	Query / Template length			

1	NODE_21_length_77330_cov_37.4653_ID_1799	IncFIA	AP001918	100	40114-40475	362 / 388			
2	NODE_21_length_77330_cov_37.4653_ID_1799	IncFIB (AP001918)	AP001918	99.71	19504-20185	682 / 682			
3	NODE_21_length_77330_cov_37.4653_ID_1799	IncFIC (FII)	AP001918	100	57552-58050	499 / 499			

C. Bacteriophage detection									

Region	JHI_5025 contig	Most Common Phage	Accession number	Score	Completeness	Region Length	Region Position	# Total Proteins	GC%

1	NODE_1_length_624063_cov_18.6826_ID_962	PHAGE_Escher_vB_EcoM_12474III	NC_049457	70	questionable	37.7Kb	7183-44933	42	50.58%
2	NODE_1_length_624063_cov_18.6826_ID_962	PHAGE_Entero_Tyrion	NC_031077	89	questionable	54.2Kb	477673-531898	60	52.51%
3	NODE_4_length_375183_cov_20.3356_ID_3592	PHAGE_Escher_SH2026Stx1	NC_049919	50	incomplete	6.7Kb	367796-374513	11	47.08%
4	NODE_5_length_327848_cov_20.9475_ID_3493	PHAGE_Shigel_SfIV	NC_022749	80	questionable	10.8Kb	23761-34590	14	44.99%
5	NODE_9_length_131940_cov_22.2706_ID_1056	PHAGE_Entero_IME10	NC_019501	110	intact	41.6Kb	202-41828	55	46.69%
6	NODE_9_length_131940_cov_22.2706_ID_1056	PHAGE_Klebsi_ST437_OXA245phi4.2	NC_049449	10	incomplete	9.4Kb	120559-130009	14	46.97%
7	NODE_20_length_81134_cov_18.2852_ID_1451	PHAGE_Klebsi_4LV2017	NC_047818	10	incomplete	11.4Kb	156-11605	18	53.45%
8	NODE_21_length_77330_cov_37.4653_ID_1799	PHAGE_Stx2_c_1717	NC_011357	70	questionable	8.3Kb	18104-26477	13	50.32%
9	NODE_24_length_65727_cov_20.6388_ID_1687	PHAGE_Entero_HK629	NC_019711	50	incomplete	7.3Kb	36313-43650	7	43.69%
10	NODE_31_length_28944_cov_23.5938_ID_1979	PHAGE_Escher_500465_1	NC_049342	60	incomplete	21.3Kb	1-21345	15	48.97%
11	NODE_35_length_9188_cov_27.7188_ID_2232	PHAGE_Entero_lambda	NC_001416	50	incomplete	9Kb	3-9078	16	44.61%
12	NODE_36_length_7654_cov_27.8584_ID_2268	PHAGE_Entero_mEp460	NC_019716	60	incomplete	7.5Kb	3-7578	16	42.29%

D. Serotype detection									

Database	Contig	Gene/Serotype	Accession number	Identity	Position in contig	Template / HSP length			

H type genes	NODE_2_length_507490_cov_20.8048_ID_781	fliC / H39	AY250019	100	150955-152253	1299 / 1299			
O type genes	…	No hit found	n/a	n/a	…	n/a			

## 2 Experimental Design, Materials and Methods

Genomic comparisons were carried out using the contig sequence fasta file for strain JHI_5025, available from ENA (project PRJEB22630, SAMEA104314548, isolated from soil, 2008, UK) and the complete sequence fasta file for reference strain MG1655 (U00096.3) using the CLC Genomics Workbench suite (Qiagen Ltd. Hilden, Germany), with the Whole Genome Alignment tool. A 2-way average nucleotide identity (ANI) was generated using an online tool [7] for positional mapping of 21,278 fragments. Unique ‘genomic island’ sequences in isolate JHI_5025 with respect to the reference sequence were identified with the Blastn algorithm [8], using default parameters (Word size, 11; Expect value, 0.05; Hitlist size, 10; Match/Mismatch scores, 2,-3; Gapcosts, 5,2; Low Complexity Filter, Yes; Filter string, L;m; Genetic Code, 1), with metrics for contiguous sequences with an arbitrary cut-off of 1000 nt in length in Table 1A. Specific sequences were detected with online tools for plasmids, bacteriophage and serotype from PlasmidFinder [9], PHASTER [10] and SeroTypeFinder [11] respectively, using default settings for each.

## Ethics Statement

n/a.

## CRediT authorship contribution statement

**Nicola Holden:** Conceptualization, Investigation, Methodology, Writing – review & editing.

## Declaration of Competing Interest

The authors declare that they have no known competing financial interests or personal relationships which have or could be perceived to have influenced the work reported in this article.
